# Chemical Composition, Antibacterial and Anti-Quorum Sensing Activities of *Pimenta dioica* L. Essential Oil and Its Major Compound (Eugenol) against Foodborne Pathogenic Bacteria

**DOI:** 10.3390/plants11040540

**Published:** 2022-02-17

**Authors:** Ayshah Aysh ALrashidi, Emira Noumi, Mejdi Snoussi, Vincenzo De Feo

**Affiliations:** 1 Department of Biology, University of Ha’il, College of Science, P.O. Box 2440, Ha’il 81451, Saudi Arabia; ayshah.a@hotmail.com (A.A.A.); snmejdi@yahoo.fr (M.S.); 2 Laboratory of Bioresources: Integrative Biology and Valorization (LR14-ES06), Higher Institute of Biotechnology of Monastir, University of Monastir, Avenue Tahar Haddad, BP 74, Monastir 5000, Tunisia; 3 Laboratory of Genetic, Biodiversity and Valorization of Bioressources, Higher Institute of Biotechnology of Monastir, University of Monastir, Avenue Tahar Haddad, BP 74, Monastir 5000, Tunisia; 4 Department of Pharmacy, University of Salerno, Via Giovanni Paolo II, 132, Fisciano, 84084 Salerno, Italy; defeo@unisa.it

**Keywords:** *Pimenta dioica*, eugenol, foodborne pathogens, biofilm, anti-quorum sensing, violacein

## Abstract

The *Pimenta dioica* essential oil and its main compound (eugenol) were tested for their antibacterial potency against eight Gram-negative and Gram-positive bacteria implicated in food intoxication. This essential oil and its main component were evaluated for their ability in inhibiting Quorum sensing (QS)-dependent mechanisms such as motility in *Pseudomonas aeruginosa* PAO1, production of violacein by *Chromobacterium violaceum* and biofilm formation on stainless steel and glass surfaces. Our results demonstrated that *P. dioica* essential oil and eugenol were active against all tested strains with a maximum of inhibition against *Listeria monocytogenes* CECT 933 (26.66 ± 0.57 mm). The minimal inhibitory concentration (MIC) value of the tested essential oil and eugenol was about 0.048 mg/mL for all strains. The obtained results demonstrated that 4CMI eugenol inhibited foodborne strains biofilm formation on the glass strips by 73.79% and by 75.90% on polystyrene. Moreover, 0.048 mg/mL (MIC) of *P. dioica* essential oil inhibited the violacein production by 69.30%. At 100 µg/mL, *P. dioica* oil and eugenol affected the motility of PAO1 by 42.00% and 29.17%, respectively. Low concentrations of *P. dioica* essential oil are active against the quorum sensing phenomena and biofilm potency. Thus, this essential oil could be further investigated for new molecules useful for the treatment of toxi-alimentary infections.

## 1. Introduction

Foodborne pathogenic bacteria are responsible of many human alimentary intoxications. The obvious examples of pathogenic bacteria are *Salmonella enterica*, *Listeria monocytogenes*, *Vibrio vulnificus*, *Shigella flexneri*, *Bacillus subtilis*, *Escherichia coli*, *Pseudomonas aeruginosa* and *Staphylococcus aureus*. These bacteria are recognized for their high ability to adhere to surfaces and epithelial cells. Food preparations are based on the use of species responsible for their aromatic properties and antimicrobial potency.

The diet based on the consumption of contaminated food with bacteria causes a big problem to public health. Several bacteria accounted for many cases of death [1]. The use of plants (spices) and natural products (herbs), which can be added during the food conception may reduce the risk of contamination with these pathogens by inhibiting their activities and food damage [2,3,4].

Essential oils are widely used for antimicrobial, anti-parasitical, insecticidal and other medicinal activities. Additionally, they are used as flavour in cosmetics, pharmaceuticals and food industries [5,6].

The *Pimenta* genus is a group of trees native throughout the Caribbean region, is recognized by its anticancer, antioxidant, antifungal, antibacterial and anti-inflammatory properties [7].

Eugenol, as an additive in alimentary industry, was the main constituent identified in *P. dioica* essential oil [3], which was recognized for its antioxidant, antimicrobial and cytotoxic activities [8,9].

Volatile oils are obtained by the process of distillation of plant parts. There are widely used for antimicrobial, antiparasitical, insecticidal and other medicinal activities. Additionally, they are used as flavours in the cosmetics, pharmaceuticals and food industries [5].

The eugenol present in *Pimenta* leaf essential oil [8], which was recognized for many activities, especially antioxidant, bactericidal and virucidal properties [9,10]. Ali et al. [11], Faria et al. [12] and many other scientists have demonstrated the inhibitory activities of *P. dioica* and eugenol against many pathogenic microorganisms such as *S. aureus*, *E. coli* and *P. aeruginosa* [13,14,15].

Several virulence factors such as adhesion and resistance to antibiotics were largely studied by scientists in order to search for a new method of therapy [16]. The formation of biofilm in bacteria strains is regulated by the mechanism of QS [16,17]. The capacity to form biofilms is considered as a QS-based factors, such as swarming, production of exopolysaccharides and inhibition of violacein production [16].

The purpose of the present study was to determine the chemical composition of *P. dioica* essential oil and to study its activity, as well as its main compound, eugenol, against several foodborne pathogenic bacteria. The anti-QS activity of *P. dioica* EO was tested using *Chromobacterium violaceum* and *Pseudomonas aeruginosa* PAO1. In addition, we reported the antibiofilm potency of different concentrations of *P. dioica* essential oil and its main component eugenol.

## 2. Results

### 2.1. Chemical Composition of P. dioica Essential Oil

**Figure 1:**
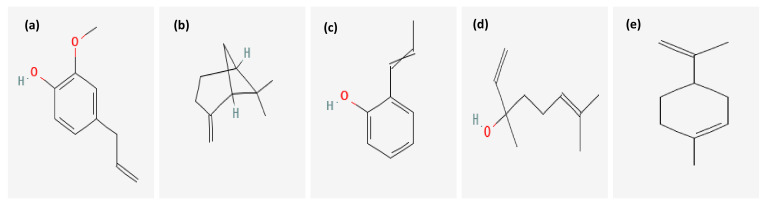
Chemical structure of the main compounds identified in *P. dioica* essential oil by GC-MS technique: (**a**) eugenol, (**b**) β-pinene, (**c**) 2-Propenylphenol, (**d**) linalool and (**e**) limonene.

The chemical composition of *P. dioica* essential oil is summarized in Table 1. Thirty components with different percentage were identified using HP5 capillary column according to their elution time. *P. dioica* essential oil was rich in eugenol (48.67%), β-pinene (18.52%) and 2-Propenylphenol (7.61%). Other relevant components were linalool (3.68%) and limonene (3.55%). The structures of the major compounds are represented in Figure 1.

### 2.2. Antibacterial Activity of P. dioica Essential Oil and Eugenol

The results demonstrated that *P. dioica* essential oil and eugenol were active against all tested strains with a maximum inhibition against *L. monocytogenes* CECT 933 (21.66 mm with *P. dioica* and 21.33 mm for eugenol). Eugenol was more active against *S. flexeneri* CECT 4804.

*P. aeruginosa* PAO1 showed a resistance to *P. dioica* and eugenol with inhibition zone diameters of 8.00 mm and 7.66 mm, respectively (Table 2).

Low concentrations of *P. dioica* essential oil and eugenol inhibited the growth of all foodborne pathogenic tested strains. In fact, the MIC value of the tested essential oil was 0.048 mg/mL for all strains. The same concentration (0.048 mg/mL) for the eugenol was able to reduce the growth of all bacterial strains. The MBC values of the tested essential oil were about 1.562 to 12.5 mg/mL and 3.125–12.5 mg/mL (eugenol) are needed to completely inhibit the growth of the Gram-positive and Gram-negative strains tested (Table 2).

### 2.3. Adhesive Properties and Biofilm Formation on Abiotic Materials

**Figure 2:**
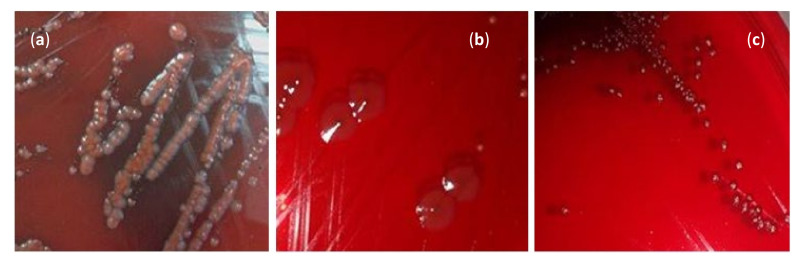
Different morphotypes of foodborne pathogenic strains cultivated on CRA: (**a**) negative morphotype, (**b**) and (**c**): positive morphotype.

Among the isolated strains, five out of eight (62.50%) were slime producing characterized by the black colonies and red with black center, and the remaining strains (*P. aeruginosa*, *B. subtilis* and *S. enterica*) were non-slime producing characterized by red bordeaux colonies (Figure 2, Table 3).

**Figure 3:**
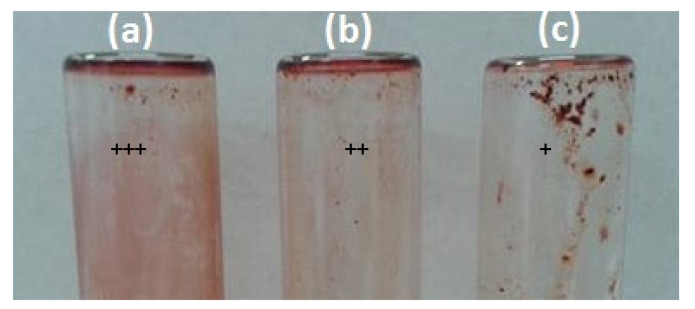
Adhesive properties on glass tube using safranin staining: (**a**) Strong adhesion (+++); (**b**) Moderate adhesion (++); (**c**) Low adhesion (+).

The study of biofilm formation on glass tubes showed that *S. flexeneri* and *L. monocytogenes* were strongly adherent (a, noted +++), and 50% of the tested strains were moderately adherent (b, noted ++) to this material (Figure 3).

**Figure 4:**
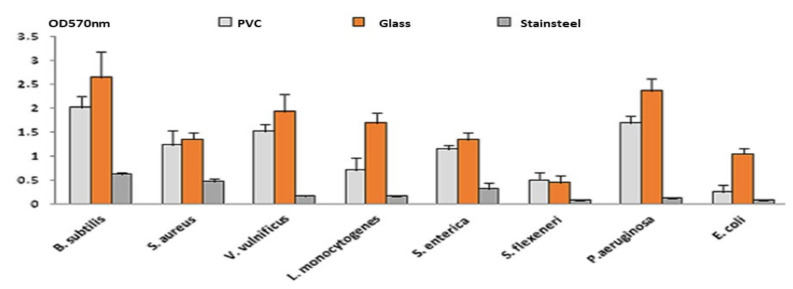
Adhesion of the selected strains to different materials (Polyvinyl chloride PVC, glass and stainless steel).

The most used materials in culinary preparations (polystyrene, glass, stainless steel and polyvinylchloride) were chosen during this work. The staining assay with 1% crystal violet (CV) showed that all foodborne pathogenic strains form a biofilm (0.1 < OD < 1 or OD_570_ > 1) on the selected materials with different degrees depending on the strain and the surface (Figure 4).

All Gram-positive and Gram-negative strains were high biofilm producers on glass with a maximum of adhesion for B. subtilis (OD = 2.65). *S. aureus* ATCC 6538 was the highest biofilm producer on polystyrene (OD > 1). *S. flexneri* CECT 4804 showed the lowest values of OD on the four tested materials (Table 3).

### 2.4. Anti-Biofilm Activity of P. dioica and Eugenol on Polystyrene and Glass Surfaces

The study of the anti-biofilm properties of *P. dioica* and eugenol was carried out on the *S. aureus* ATCC 6538 strain according to its high potency of biofilm formation.

*P. dioica* showed an anti-biofilm ability of the *S. aureus* strain on glass and polystyrene about 55% and 58%, respectively, at the lowest tested concentration (MIC = 0.048 mg/mL) (Table 4). This effect was stronger when we tested the anti-biofilm effect of eugenol against the same strain showing an inhibition about 73.00% on polystyrene. In fact, low concentrations (0.048, 0.096 and 0.192 mg/mL) of this compound demonstrated an important reduction of the biofilm formation on both tested surfaces.

The main compound, eugenol, was more active on sessile *S. aureus* ATCC 6538 isolate adherent into polystyrene and glass than the essential oil. The effect of *P. dioica* essential oil and eugenol on biofilm formed on polystyrene and glass was not variable depending on the concentration and tested material. A 4 × MIC concentration of *P. dioica* EO inhibited the *S. aureus* biofilm formed on glass and polystyrene with percentages of (64.41 ± 1.4%) and (70.25 ± 1.19%), respectively. All these results are summarized in Table 4.

### 2.5. Violacein Inhibition Assay in C. violaceum

In qualitative analysis, *P. dioica* essential oil inhibited the production of violacein in *C. violaceum* ATCC 12472 with a percentage of inhibition more than 50% (71.30 ± 1.5%) at MIC value even at a low concentration (MIC/4) (Table 5).

**Figure 5:**
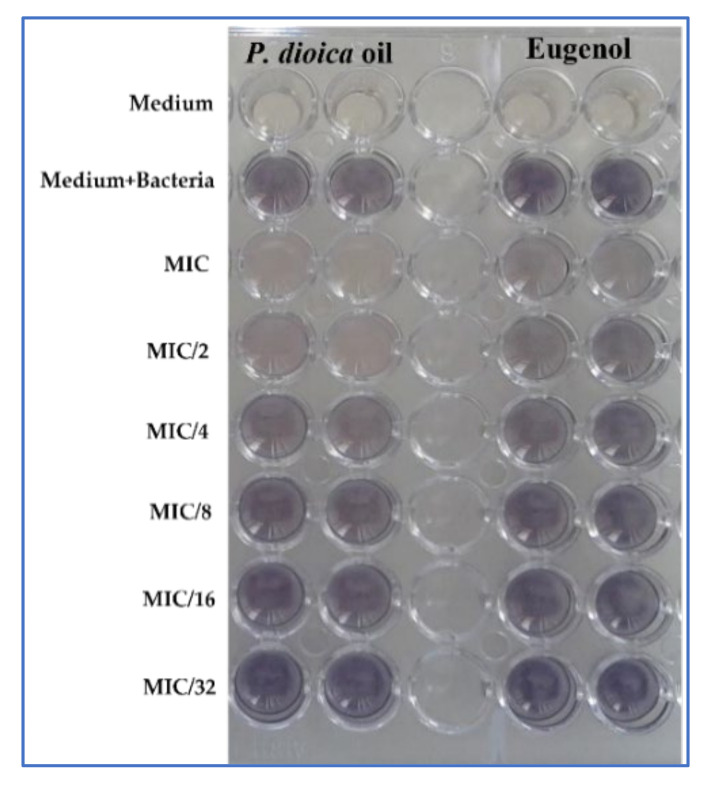
Effects of different MIC values of *P. dioica* essential oil and eugenol on violacein inhibition (qualitative method with *C. violaceum* ATCC 12472).

However, violacein production was inhibited only to an extent of 48.29 ± 0.9% when we tested the eugenol (Table 5, Figure 5).

### 2.6. Anti-Swarming Assay

During this essay, we examined the anti-QS potential of *P. dioica* essential oil and eugenol on swarming motility in PAO1 strain. The results indicated that *P. dioica* essential oil and its main compound inhibited the swarming of PAO1 to different extents and at the selected doses (50, 75 and 100 µg/mL). Moreover, 100 µg/mL were able to inhibit swarming about 42.00% and 29.00% for *P. dioica* oil and eugenol, respectively (Table 6).

## 3. Discussion

*P. dioica* has been used as an important spice for its culinary and medicinal uses [18]. This plant was used to reduce muscle pain, help digestion and stomach gases [19,20]. In Cuba, this species can be cooked or ingested to treat stomach pain and colds [21]. The leaves of *Pimenta* are used to reduce arthritis, fever and stress and in India [22].

Many compounds have been isolated from this plant such as tannins, glycosides, phenylpropanoids and essential oils [23].

Many constituents found in *P. dioica* berries and leaves such as galloylglucosides phenylpropanoids [24], tannins and flavonoids [25] showed several properties (antibacterial, analgesic hypotensive and anti-neuralgic). Considering the phytochemical composition and low cost of *Pimenta* berries, this spice may be used in the food preparation [26] (Table 7).

Eugenol represents the main compound of the oil (48.67%). Our results are similar to previous literature [24]. These researchers identified 22 compounds in *Pimenta* oils. The eugenol was the main component of both oils with 71.40% in leaves and 65.90% in fruits [27].

Our study showed that the tested *P. dioica* essential oil was rich in eugenol (48.67%), β-pinene (18.52%) and (1E)-Phenol-2-propenyl (7.61%). Other relevant components were linalool (3.68%) and limonene (3.55%) (Table 7).

The essential oil of *P. dioica* fruits from Jamaica was composed of eugenol essentially (68–78%) [33]. In addition, the volatile oil from *P. dioica* fruits originating from Mexico was rich in eugenol (90%) and α-terpineol (2%) [34]. The same result was obtained in Guatemalan fruits [35].

*P. dioica* has been described for several years for its biological uses. Our results demonstrated that its essential oil and eugenol were active against all tested foodborne pathogenic strains. The essential oil of *P. dioica* berries inhibited *L. monocytogenes*, *Salmonella* typhimurium, *Pseudomonas putida*, *E. coli* and *S. aureus* [36]. The essential oil of the same plant extracted from its leaves demonstrated a strong antibacterial activity against *Pseudomonas* and *Staphylococcus* species [13].

The antimicrobial activity of *P. dioica* fruits and leaves extracts and essential oil has been proved. *P. dioica* leaf extracts presented significant antimicrobial properties against many genera of bacteria and fungi such as *Escherichia coli*, *Streptococcus mutans*, *Staphylococcus aureus*, *Bacillus cereus*, *Pseudomonas fluorescens*, *Salmonella* typhimurium, *Candida albicans*, *Aspergillus niger* and *Penicillium* sp. [37,38,39].

Mérida-Reyes et al. [27] tested the antibacterial activity of the essential oil of leaves of *P. dioica* against *B. subtilis*, *S. aureus*, *S. enterica* and *E. coli*. Their results showed that this oil is very active against *B. subtlis* and *E. coli* was the more resistant strain. The activity of the oil against bacteria depends on the synthesis of the cell wall interfering in the formation of the peptidoglycan molecule [40]. Some authors related to this antibacterial activity to the presence of eugenol and (E)-caryophyllene since these two compounds were currently found in *P. dioica* oil [40,41]. In addition, it has been demonstrated that eugenol is active on the cytoplasmic membrane [30].

It has been demonstrated that eugenol can reduce the production of pyocyanin and biofilm formation in *E. coli* and *S. aureus* [42].

Using the CRA test, 62.50% were slime-producing, characterized by a black colonies and red with black centers. Pigmented colonies were considered as slime-producing strains, whereas unpigmented colonies were classified as non-slime-producing strains [43].

Many works have proved the anti-biofilm potency of monoterpenoids on Gram-negative and Gram-positive bacteria during the biofilm development [44].

Eugenol is largely used as a flavoring agent in the food industry due to its biological properties such as anti-inflammatory, anti-microbial and antioxidant. This compound is used against Gram-positive and Gram-negative bacteria. It is demonstrated that eugenol presents strong inhibition against several anaerobic bacteria (*Streptococcus mutans* and *Prevotella intermedia*), *Listeria monocytogenes* and *Candida albicans*. Several essential oils are active against bacterial biofilm development such as clove and pimento berry oil [45]. This compound inhibits QS of *P. aeruginosa* [46]. Some studies reported that thymol and carvacrol were responsible for the anti-QS activity [47].

Burt (2004) [48] demonstrated that essential oils containing phenolic compounds such as eugenol thymol or carvacrol have the strongest antimicrobial activity. Gram-negative bacteria are known to be more resistant to volatile oils than the Gram-positive bacteria [48]. The same scientists proved that monoterpenes (limonene and α-pinene) inhibited biofilm formation more than terpene alcohols such as linalool and terpinene-4-ol. The most foodborne pathogenic bacteria such as the *Pseudomonas* produce biofilms [49]. The family *Lamiaceae* is considered as important aromatic plants with constituents having anti-QS properties to combat different food pathogenic microorganisms [50]. Other studies demonstrated that single constituents of essential oils such as eugenol, linalool, γ-terpinene and limonene exhibited anti-QS effects [51].

## 4. Materials and Methods

### 4.1. Bacterial Strains

The antibacterial effect of the volatile oil of *P. dioica* and its main component the eugenol was tested against eight food-borne pathogenic bacteria including four Gram-positive (*Staphylococcus aureus* ATCC 6538, *Bacillus subtilis* CIP 5265, *Vibrio vulnificus* CECT 529, *Listeria monocytogenes* CECT 933) and four Gram-negative bacterial strains (*Pseudomonas aeruginosa* PAO1, *Escherichia coli* ATCC 35218, *Salmonella enterica* CECT 443, *Shigella flexeneri* CECT 4804) were procured from American Type Culture Collection (ATCC) USA and Spanish Type Culture Collection (CECT).

### 4.2. Chemical Characterization of the Essential Oil

*P. dioica* essential oil was purchased from Huile & Sens (Crestet, France) on 27 November 2014 (Product number B750N06). This oil was extracted from the dried unripe fruits by hydrodistillation technique. The main compound (eugenol) was purchased from Sigma (Sigma-Aldrich S.r.l. Milan, Italy). The essential oil was analyzed by gas chromatography–flame ionization detector (GC–FID) and gas chromatography–mass spectrometry (GC–MS) [52,53,54,55] and mass spectra on both columns with those of authentic compounds available in our laboratories by means NIST 02 and Wiley 275 libraries [56].

### 4.3. Antimicrobial Activities

#### 4.3.1. Disk-Diffusion Assay

Antimicrobial activity testing was performed according to the protocol described by Noumi et al. [57]. Bacterial strains were enriched on a tube containing 9 mL of Mueller–Hinton (MH) broth then incubated at 37 °C for 24 h. The inoculums were streaked onto Mueller–Hinton agar plates using a sterile swab. Tetracycline was used in this study as positive control. The antibiotic susceptibility was determined by using the Kirby–Bauer method and Mueller–Hinton agar plates.

#### 4.3.2. Microdilution Method for the Determination of the MIC and MBC

The MIC and the MBC values were determined for all bacteria as described by Hajlaoui et al. [58]. The inoculums of the bacterial strains were prepared from an overnight broth cultures (37 °C) and suspensions and were adjusted to OD_600_ (10^7^ CFU/mL). The essential oil dissolved in 10% dimethylsulfoxide (DMSO) with a high concentration about 50 mg/mL. Serial two-fold dilutions of the stock solution of the essential oil were prepared in 96-wells plate containing 95 µL of Mueller–Hinton broth for bacteria. In fact, 100 µL aliquot from the stock solution (50 mg/mL) was added to the first well containing 95 µL of the correspondent broth. Then, a serial two-fold dilutions was prepared by transferring 100 µL from the first well into the 10 consecutive wells. The last well containing 195 µL of Mueller–Hinton broth without essential oil and 5 µL of the inoculum on each strain was used as the negative control. Finally, 5µL of the inoculum of each microorganism was added to the wells with a final volume about 200 µL in each well. We have used the scheme proposed for essential oils by Aligiannis et al. [52]: strong activity (0.05 < MIC < 0.5 mg/mL), moderate activity (0.6 < MIC < 1.5 mg/mL) and weak activity (MIC > 1.5 mg/mL).

### 4.4. Biofilm Formation Ability of Tested Isolates

#### 4.4.1. Phenotypic Characterization of Bacteria-Producing Slime

Detection of slime producing strains was carried out by culturing the isolates on Congo Red Agar (CRA) plates as previously described by Touati et al. [53]. The plates were prepared by mixing 36 g of saccharose (Sigma Chemical Company, St. Louis, MO, USA) with 0.8 g of Congo red in 1 L of Brain Heart Infusion (BHI) agar (Biorad, Hercules, CA, USA). After incubation for 24 h at 37 °C, black colonies and colonies red with a black center were considered as positive slime producers [53].

#### 4.4.2. Test Tube Method

Slime production on glass tubes was determined using the Safranin staining as described for coagulase negative staphylococci by incubating bacterial culture into a glace test tube containing 10 mL of LB broth supplemented with 8% of glucose [54]. Slime production was interpreted as negative, weak (1+), moderate (2+) or strong (3+).

#### 4.4.3. Biofilm Formation in 96-well Polystyrene Plates and Glass

Biofilm production by foodborne pathogenic bacterial strains grown in BHI (Bio-Rad, France) was assessed using crystal violet staining assay [55]. Biofilm formation was interpreted as highly positive (OD_570_ ≥ 1), low grade positive (0.1 ≤ OD_570_ < 1) or negative (OD_570_ < 0.1).

For biofilm formation on glass, the strips (1.5 cm^2^) were disinfected by dipping in 70% alcohol for 30 min and was held with sterile distilled water. Biofilm quantification was made with crystal violet 1% staining. Moreover, 125 µL of each well were transferred on 96-well microtiter plate and the OD at 570 nm was measured [16].

### 4.5. Determination of Anti-Biofilm Activity on Polystyrene and Glass

MIC, 2 × MIC and 4 × MIC of *P. dioica* essential oil and eugenol were tested for their anti-*Staphylococcus* biofilm formation. Only *S. aureus* ATCC 6538 strain was selected for this test. The crystal violet staining was employed to test the effects on biofilm formation. One hundred µL of fresh bacterial suspension was added to each well. Growth control, media control and blank control were included. The biofilm formation was evaluated using the crystal violet staining method as described previously [16,57].

### 4.6. Violacein Inhibition Assay

Various concentrations of *P. dioica* essential oil and eugenol (MIC = 10 mg/mL until MIC/32 = 0.3125 mg/mL) were added to 10 µL of *C. violaceum* ATCC 12472 and incubated at 30 °C for 18 h for the qualitative screening of violacein inhibition [57,59].

### 4.7. Swarming Assay

*P. aeruginosa* PAO1 strain were point inoculated on plates (1% peptone, 0.5% Na Cl, 0.5% agar and 0.5% D-glucose) with various concentrations of *P. dioica* essential oil and eugenol (50, 75 and 100 µg/mL) [59,60].

### 4.8. Statistical Analysis

All the experiments were conducted in triplicate and average values were calculated using the SPSS 16.0 statistics package for Windows. The differences in mean were calculated using the Duncan’s multiple-range tests for means with 95% confidence limit (*p* ≤ 0.05). Values were expressed as means ± standard deviations.

## 5. Conclusions

In this work, we reported the isolation of eugenol (48.76%) and β-pinene (18.52%) as the main phytocompounds in *P. dioica* essential oil. *Shigella*, *Vibrio*, *Listeria*, *Bacillus*, *Salmonella*, *Escherichia*, *Pseudomonas* and *Staphylococcus* foodborne pathogenic bacteria were highly sensitive to the tested oil with mean diameter of growth inhibition zone ranging from (8.00 ± 0.01) mm to (26.66 ± 0.57) mm. Low concentrations of *P. dioica* essential oil and eugenol were necessary to inhibit the growth of all tested microorganisms. While concentrations as low as 12.5 mg/mL for *Pimenta* essential oil and 3.125 mg/mL for eugenol are needed to kill the tested strains. Additionally, *P. dioica* essential oil and eugenol were able to inhibit the biofilm formation on abiotic surfaces (Polystyrene and glass) by almost all tested foodborne strains. Moreover, the tested essential oil and eugenol were able to regulate the production of some virulence related properties controlled by the quorum sensing mechanism in *C. violaceum* and *P. aeruginosa* PAO1 starter strains. Hence, these findings highlighted the potential use of this essential oil as a potential candidate for food preservation, biofilm prevention and bacterial cell to cell communication inhibitor.

## Figures and Tables

**Table 1:** GC-MS results showing the chemical composition of *P. dioica* essential oil.

N.	Compound	Ki ^a^	Ki ^b^	% ^c^
1	α-thujene	916	930	0.05
2	α-Pinene	924	939	0.42
3	1-Octen-3-ol	970	979	1.42
4	3-Octanone	977	983	0.42
5	β-Pinene	983	979	18.52
6	3-Octanol	986	991	0.47
7	δ-2-carene	992	1002	0.52
8	δ-3-carene	1004	1011	0.21
9	ρ-Cymene	1012	1024	0.84
10	Limonene	1017	1029	3.55
11	(Z)-β-Ocimene	1038	1037	0.49
12	γ-Terpinene	1048	1059	0.06
13	Benzyl Formate	1077	1076	0.18
14	Linalool	1091	1096	3.68
15	Isopulegol (neoiso)	1167	1171	0.61
16	α-terpineol	1180	1188	0.17
17	Methyl chavicol	1187	1196	0.47
18	2-Propenylphenol	1247	1267	7.61
19	5-Indanol	1336	1341	0.12
20	Eugenol	1356	1359	48.67
21	α-copaene	1367	1376	0.29
22	α-gurjunene	1409	1409	0.93
23	α-muurolene	1443	1454	0.26
24	9-*epi*-(E) Caryophyllene	1466	1466	0.26
25	Germacrene D	1485	1485	0.10
26	γ-amorphene	1497	1495	0.21
27	γ-patchoulene	1503	1502	0.17
28	δ-amorphene	1513	1512	0.74
29	γ–eudesmol	1631	1631	0.18
30	α-muurolol	1644	1646	0.16

^a^ Kovats retention index determined relatively to the tR of a series of n-alkanes (C10–C35) on HP-5 MS column. ^b^ Kovats retention index determined relatively to the tR of a series of n-alkanes (C10–C35) on HP Innowax. ^c^ t = trace (<0.1%).

**Table 2:** Antibacterial activity of *P. dioica* essential oil and eugenol against several foodborne pathogenic bacteria.

Strains	*P. dioica* EO			Eugenol		
	IZ (mm ± SD)	MIC (mg/mL)	MBC (mg/mL)	IZ (mm ± SD)	MIC (mg/mL)	MBC (mg/mL)
*Listeria monocytogenes* CECT 933	26.66 ± 0.57 ^a^	0.048	12.5	21.33 ± 0.57 ^A^	0.048	3.125
*Vibrio vulnificus* CECT 529	19.00 ± 0.01 ^d^	0.048	12.5	17.66 ± 0.57 ^B^	0.048	12.5
*Shigella flexeneri* CECT 4804	17.00 ± 0.01 ^e^	0.048	3.125	18.66 ± 0.57 ^B^	0.048	12.5
*Bacillus subtilis* CIP 5265	22.33 ± 0.57 ^c^	0.048	12.5	6.00 ± 0.01 ^E^	0.048	3.125
*Salmonella enterica* CECT 443	24.33 ± 0.57 ^b^	0.048	3.125	22.00 ± 0.01 ^A^	0.048	3.125
*Escherichia coli* ATCC 35218	16.67 ± 0.57 ^e^	0.048	3.125	14.33 ± 0.81 ^C^	0.048	3.125
*Pseudomonas aeruginosa* PAO1	8.00 ± 0.01 ^f^	0.048	12.5	7.66 ± 0.57 ^D^	0.048	12.5
*Staphylococcus aureus* ATCC 6538	17.00 ± 1.00 ^e^	0.048	1.562	18.00 ± 1.00 ^B^	0.048	3.125

IZ: Inhibition zone; MIC: Minimal inhibitory concentration; MBC: Minimal bactericidal concentration; SD: Standard deviation. The letters (a–f) and (A–E) indicate a significant difference according to Duncan test (*p* < 0.05).

**Table 3:** Slime production, qualitative and quantitative adhesive properties of selected strains on glass and polystyrene.

Strains	Adhesion to Glass	Slime Production on CRA		Adhesion to Polystyrene	
		Colour	S+/S−	OD_570_ ± SD	Production of Biofilm
*S*. *aureus* ATCC 6538	++	Black	S+	1.36 ± 0.20	High productrice
*P. aeruginosa* PAO1	++	Red bordeaux	S−	0.42 ± 0.26	Low productrice
*E*. *coli* ATCC 35218	++	Redwith black center	S+	0.17 ± 0.03	Low productrice
*S. flexeneri* CECT 4804	+++	Redwith black center	S+	0.10 ± 0.01	Low productrice
*B*. *subtilis* CIP 5265	+	Red bordeaux	S−	0.12 ± 0.01	Low productrice
*V*. *vulnificus* CECT 529	++	Redwith black center	S+	0.13 ± 0.02	Low productrice
*S*. *enterica* CECT 443	+	Red bordeaux	S−	0.15 ± 0.01	Low productrice
*L*. *monocytogenes* CECT 933	+++	Red with black center	S+	0.19 ± 0.07	Low productrice

OD: Optical density; SD: Standard deviation; +: low adhesion; ++: moderate adhesion; +++: High adhesion; S+: Slime producer; S−: Non slime producer.

**Table 4:** Effect of MIC, 2MIC and 4MIC of *P. dioica* essential oil and eugenol on *S. aureus* ATCC 6538 biofilm formed on polystyrene and glass.

Essential Oil/ Main Compound	Percentage of Inhibition of *S. aureus* ATCC 6538 Biofilm Formed on					
		Polystyrene	Glass			
	MIC	2xMIC	4xMIC	MIC	2xMIC	4xMIC
***P. dioica***	55.05 ± 3.23 ^b^	60.66 ± 1.01 ^a^	64.41± 1.4 ^a^	58.01 ± 1.62 ^c^	63.01 ± 0.53 ^b^	70.25 ± 1.19 ^a^
**Eugenol**	73.25 ± 2.68 ^A^	75.31 ± 2.02 ^A^	75.90 ± 1.76 ^A^	67.25 ± 0.68 ^B^	72.75 ± 0.92 ^A^	73.79 ± 1.47 ^A^

MIC: Minimal inhibitory concentration; MIC of *P. dioica* = 0.048 mg/mL; MIC of eugenol = 0.048 mg/mL. The letters (a–c) and (A, B) indicate a significant difference according to Duncan test (*p* < 0.05).

**Table 5:** Percentage of violacein inhibition using *C. violaceum* ATCC 12472 strain.

Concentration	% of Violacein Inhibition	
	*P. dioica*	Eugenol
MIC	71.30 ± 1.5 ^a^	48.29 ± 0.9 ^a^
MIC/2	67.87 ± 1.7 ^b^	37. 78 ± 1.8 ^b^
MIC/4	55.74 ± 0.71 ^c^	33.91 ± 1.1 ^c^
MIC/8	38.25 ± 1.8 ^d^	0 ± 1.5 ^d^
MIC/16	32.34 ± 1.3 ^e^	6.65 ± 0.7 ^e^
MIC/32	17.65 ± 0.7 ^f^	3.41 ± 1.1 ^f^

The letters (a–f) indicate a significant difference according to Duncan test (*p* < 0.05).

**Table 6:** Effect of 50, 75 and 100 µg/mL of *P. dioica* essential oil and eugenol on swarming motility of PAO1.

Component	Concentration		
	50 µg/mL	75 µg/mL	100 µg/mL
*P. dioica*	17 ± 0 ^c^	25 ± 0 ^b^	42 ± 0 ^a^
Eugenol	20.83 ± 1.17 ^B^	29.17 ± 0.17 ^A^	29.17 ± 0.17 ^A^

The letters (a–c) and (A, B) indicate a significant difference according to Duncan test (*p* < 0.05).

**Table 7:** Chemical composition of essential oils of the genus *Pimenta* function of origin and plant organ.

Plant Species (Origin)	Organ	Extraction Method	Main Compounds	Reference
*P. adenoclada* (Cuba)	Leaves	Hydrodistillation	Caryophyllene oxide (15.4), α-muurolol (9.4), humulene epoxide II (7.6), trans-sabinol (5.6), β-pinene (5.3)	[28]
*P. dioica* (Jamaica)	Leaves	Steam distillation	Eugenol (66.38–79.24), β-caryophyllene (0.97–7.10)	[29]
*P. dioica* (México)	Berries	Steam distillation	Methyl-eugenol (48.3), myrcene (17.7), eugenol (17.3), β-caryophyllene (6.2)	[30]
*P. dioica* (Australia)	Leaves	Supercritical CO_2_	Eugenol (77.9), β-caryophyllene (5.1), squalene (4.1)	[31]
*P. dioica* (Brazil)	Fruits	Hydrodistillation	Eugenol (76.98), β-pinene (6.52), limonene (4.09)	[32]
*P. dioica*	Fruits	Hydrodistillation	Eugenol (48.67%), β-pinene (18.52%), (1E)-Phenol-2-propenyl (7.61%)	This study

## Data Availability

The data generated and analyzed during this study are included in this article.
